# On-Demand Anonymous Access and Roaming Authentication Protocols for 6G Satellite–Ground Integrated Networks

**DOI:** 10.3390/s23115075

**Published:** 2023-05-25

**Authors:** Ya Tao, Haitao Du, Jie Xu, Li Su, Baojiang Cui

**Affiliations:** 1 School of Cyberspace Security, Beijing University of Posts and Telecommunications, Beijing 100876, China; taoya@bupt.edu.cn (Y.T.); cuibj@bupt.edu.cn (B.C.); 2 China Mobile Research Institute, Beijing 100032, China; duhaitao@chinamobile.com (H.D.); suli@chinamobile.com (L.S.)

**Keywords:** satellite–ground integrated network, 6G, privacy preserving, authentication protocol

## Abstract

Satellite–ground integrated networks (SGIN) are in line with 6th generation wireless network technology (6G) requirements. However, security and privacy issues are challenging with heterogeneous networks. Specifically, although 5G authentication and key agreement (AKA) protects terminal anonymity, privacy preserving authentication protocols are still important in satellite networks. Meanwhile, 6G will have a large number of nodes with low energy consumption. The balance between security and performance needs to be investigated. Furthermore, 6G networks will likely belong to different operators. How to optimize the repeated authentication during roaming between different networks is also a key issue. To address these challenges, on-demand anonymous access and novel roaming authentication protocols are presented in this paper. Ordinary nodes implement unlinkable authentication by adopting a bilinear pairing-based short group signature algorithm. When low-energy nodes achieve fast authentication by utilizing the proposed lightweight batch authentication protocol, which can protect malicious nodes from DoS attacks. An efficient cross-domain roaming authentication protocol, which allows terminals to quickly connect to different operator networks, is designed to reduce the authentication delay. The security of our scheme is verified through formal and informal security analysis. Finally, the performance analysis results show that our scheme is feasible.

## 1. Introduction

The fifth generation (5G) of wireless communication technology has promoted the development of the Internet of Things, automatic driving, virtual reality [1], etc. However, challenges still exist [2,3]. Although the terrestrial network has developed unprecedentedly, the seamless coverage of global heterogeneous networks has not been achieved. It is difficult for users to enjoy high-quality network services in many underserved areas (such as mountains, oceans, etc.) and in more than 50% of countries [4]. Oriented toward the 6G network, satellite–ground integrated networks (SGIN), which could provide global coverage and integrated management of satellite and terrestrial networks, have become a hot topic of current research [5,6,7]. In recent years, Amazon, SpaceX, and other major manufacturers are making great efforts to build satellite networks, which are expected to form a satellite communication system with a network capacity of 10 Tbps [8], providing reliable communication services for SGIN. With 6G, satellites will obtain more computing power through mobile edge computing (MEC) [9] and they will therefore be able to undertake heavier computing tasks.

Nevertheless, due to the high cost of satellite launches and maintenance, it is difficult for large satellite operators to hold all the satellites. There will still be many satellite operators maintaining a small number of satellites and providing personalized services. Owing to the short coverage time per satellite, users may need to switch operators or switch to a faster terrestrial network. In order to support global coverage, it is important to encourage operators of SGINs to collaborate with each other and provide cross-domain services. In a word, a SGIN is a complex heterogeneous network that combines multiple operators with global coverage.

**Figure 1:**
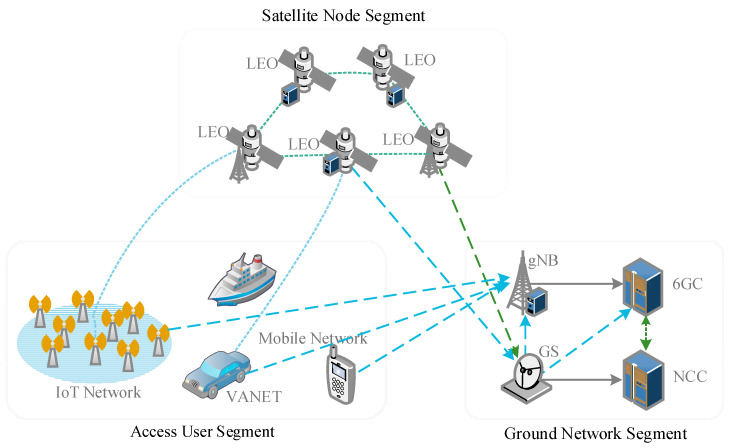
SGIN overall architecture.

As shown in Figure 1, the SGIN architecture consists of three segments, i.e., a Satellite Node Segment, Ground Network Segment, and Access User Segment. The Satellite Node Segment consists of geosynchronous earth orbit (GEO) satellites, medium earth orbit (MEO) satellites, and low earth orbit (LEO) satellites. Compared to MEOs and GEOs, LEOs are closer to the ground and therefore have less communication overheads. Thus, only LEO authentication is considered in our architecture. These satellites can be transparent satellites or regenerative satellites. Transparent satellites are only responsible for transmitting messages. While regenerative satellites, which are equipped with gNB-DU, can be applied as a part of a 6G base station to process messages. The Ground Network Segment includes heterogeneous networks, such as large-scale servers, base stations, and satellite ground stations (GSs). Among them, the satellite network operators maintain the satellite ground network, including GSs and network control centers (NCCs). The terrestrial mobile operators maintain terrestrial networks, including the next-generation NodeBs (gNBs) and 6G core networks (6GCs). The Access User Segment consists of the user equipment (UE) in heterogeneous networks, such as mobile UE, IoT UE, and marine UE, which can be located on the ground, in suburban areas (areas with poor signal), at sea, in mountains, and so on. Obviously, these devices have different computing capabilities and diverse security requirements. They should choose suitable network domains and operators according to their own needs.

Since the information in a SGIN is transmitted through a public wireless channel, the user’s information is vulnerable to malicious attacks. Access authentication is the first step for UE to connect to the core network, so as to guarantee mutual authentication, forward and backward security, and be resistant to typical attacks. Unlike existing terrestrial networks, there is a large propagation delay (more than 10 ms) between satellite and terrestrial networks. In the traditional satellite network authentication scheme, the satellite generally transmits the forwarding messages [10], which causes a transmission delay in communication at least four times that of satellite and terrestrial networks [11]. Therefore, addressing efficient and secure authentication in SGINs is the first issue.

To make things even more challenging, heterogeneous devices need to be authenticated in this network. Based on our above architecture, the different types of user equipment and their diverse security requirements pose great challenges to the research of SGIN access authentication. At present, terrestrial networks usually use the authentication and key agreement (AKA) protocol to implement UE access authentication. The 5G AKA proposes to protect the privacy of users by encrypting their permanent identity and transmitting encrypted SUCI. However, there is no privacy authentication for heterogeneous terminals that access via satellite networks. For diverse users in SGIN, a targeted authentication model is required. For example, for IoT nodes with a large scale and relatively weak computing power, anonymous batch authentication with lower complexity is needed to achieve a faster speed. However, the existing batch authentication schemes do not effectively protect user privacy [11] or incur excessive costs [12]. While individual end-users are more computationally capable and care a lot about their privacy and security, they can choose protocols with higher security and better protect their privacy. That is to say, it is difficult to unify these users with different needs.

Meanwhile, due to the existence of multiple operators, SGIN secure roaming authentication also deserves attention. The current situation of many large satellite service providers (such as SpaceX and OneWeb) [13,14] as well as small providers cannot achieve continuous global coverage of signals, which causes many inconveniences for UE to enjoy diverse and stable services. As mentioned above, different network services in 6G may be provided by various operators, and the core network (CN) of satellite and terrestrial networks may be disparate. If a new satellite or terrestrial base station providing service to a subscriber originates from a different operator, the new operator needs to perform roaming authentication with the subscriber. However, the existing 3GPP roaming authentication approach [15] is not applicable for SGINs, because satellite transmission will bring significant time delays. How to provide fast multi-operator cross-network roaming authentication for subscribers is also a key challenge.

Motivated by these challenges, we present an on-demand authentication protocol model for SGINs. In the model, we propose protocols for mutual authentication of UE and satellites, to reduce transmission overheads. Our protocols propose different access authentication scenarios for different users’ performance and security requirements, so that UE can have on-demand access. Among these, UE privacy is protected to different degrees. In addition, a roaming authentication protocol is proposed for cross-domain roaming by different operators, in line with the mutual authentication between the UE and satellite. Compared with other related works, we give consideration to authentication and roaming, and provide on-demand access authentication for terminals with different abilities and needs. The contributions of this paper are as follows:

- On-demand privacy-preserving authentication protocols for SGIN: We propose an on-demand access authentication protocol for satellite networks in SGINs based on the protocol architecture. For UE with high security and privacy requirements, an anonymous unlinkable authentication protocol is proposed which ensures UE’s unlinkability. For large numbers of UE with demand for short delay times, a batch authentication protocol is proposed. The protocol supports rebatch authentication after authentication failure and can effectively alleviate DoS attacks.
- A lightweight roaming authentication protocol for SGIN: The roaming authentication protocol provides a strategy for roaming between different operator networks for satellite-connected UE in SGINs, which needs to pre-negotiate with the corresponding core network after the last authentication is completed. The UE only needs to complete mutual authentication with the satellite when roaming, thus reducing the propagation delay.

The remainder of this article is organized as follows: We review the related work in Section 2. Then, we introduce the prior knowledge involved in the protocol and SGIN system model in Section 3. The details of our scheme are presented in Section 4. The security of the proposed scheme is proven in Section 5. We compare the performance of related schemes in Section 6. Finally, we summarize the article in Section 7.

## 2. Related Work

In recent years, researchers have made many contributions to the access authentication of SGINs. Nguyen et al. [2] provided a systematic overview of 6G security and privacy issues. They analyzed the security architecture of 6G and considered the new open authentication protocols (e.g., satellite, sea area) for non-3GPP networks, as one of the priorities for 6G network access security. Zhao et al. [16] made use of the broadcasting function of satellites to propose an efficient and lightweight access authentication scheme, to prevent the burden of “message storms” on satellite authentication. Cui et al. [17] proposed an authentication scheme for heterogeneous B5G networks (including satellite networks) and proposed a user detection scheme based on trust evaluation. Guo et al. [18] proposed an anonymous mutual authentication scheme based on RLWE, which can resist attacks based on quantum computing and guarantee efficiency and security in the post-quantum era. Yao et al. [19] proposed a mutual authentication protocol named IMAS, which introduced group management forms to the satellite, to accomplish the multicast authentication between UE and satellites. Guo et al. [20] proposed an access authentication protocol based on elliptic curve cryptography (ECC), which included three entities: the UE, satellite, and ground station. In addition, the scheme designed a batch handover scheme to reduce overheads.

In the face of the high privacy requirements of users of 6G, anonymous authentication based on aliases or group signature authentication can be used. Although the alias mechanism [21] has good performance in information transmission and privacy protection, users need to store a large number of certificates, which leads to a large amount of overheads [22]. The traditional group signature message will bring some transmission overheads. Boneh et al. [23] proposed a short group signature (SGS) scheme, which allows bilinear pairing to be widely used in modern cryptography. Wasef and Shen [12] proposed a batch authentication scheme based on SGS, so that SGS can be applied to a large number of user authentication scenarios. Alamer [24] proposed a scheme to transform the SGS signature algorithm into a signcryption algorithm, which ensures message integrity and confidentiality.

Owing to the large number of user nodes, researchers have proposed batch authentication. Huang et al. [25] proposed a fast anonymous batch authentication scheme for vehicle networking, which can verify multiple requests at a time and negotiate a session key with the vehicle through broadcast messages. Considering that a failure of batch authentication will lead to the failure of all authentication of a batch of nodes, the author also proposed to rebatch authentication to prevent possible DoS attacks. Lai et al. [26] proposed a lightweight group authentication scheme for M2M networks, and the UE in their scheme can also accomplish rebatch authentication by dichotomizing. Mahmood et al. [27] proposed ECC-based lightweight security without using a batch verification method (LSWBVM). Their method can authenticate a large number of request messages and verify messages one by one.

Due to the presence of multiple SGIN operators, cross-domain roaming authentication has become a research direction. Xue et al. [28] proposed a lightweight group key negotiation protocol based on $\left(t,\;n\right)$ secret sharing and proposed a cross-domain handover authentication scheme. Considering the problems of different operators in the converged network, Liu et al. [29] proposed a decentralized anonymous authentication scheme applied to the cross-operator satellite service scenario, which can carry out cross-domain fast handover authentication and ensure the fairness of billing. Yang et al. [30] proposed an authentication scheme based on group signatures and completed cross-domain roaming of users through advanced negotiation between ground stations and satellites. Guo et al. [31] proposed a new secure roaming authentication and key negotiation protocol called SRAKN, which enables efficient and fast roaming between users, satellites, and foreign terrestrial control stations (FTCS), and finally negotiates secure session keys. Yang et al. [32] proposed a fast handover authentication protocol for high-speed mobile terminals for railways in SGINs. Their method forms a temporary group of terminals in the same compartment and completes the handover based on preset information. Table 1 shows a summary of the related works described in this paper. We study their schemes according to four aspects of these related works: performance objective, algorithm/scheme, scenario, and motivation.

## 3. Preliminaries and System Model

In this section, we first review the mathematical preliminaries of our scheme, including bilinear pairing and ECDSA. Then, we present the system model and security requirements for SGIN networks. In particular, we describe the model of the SGIN protocol in detail. We describe the security model adopted by the SGIN system and provide security requirements to meet the security assumptions.

### 3.1. Bilinear Pairing

Let G be the addictive cyclic group of the prime order q and $G_{T}$ be the multiplicative cyclic group of the same prime order q. Let P be a generator of group of G. The bilinear pairing $\hat{e}:G\times G\to G_{T}$ satisfies:

- Bilinearity: $\hat{e}\left(aP,bQ\right)=\hat{e}{\left(P,Q\right)}^{ab}$, where $a,b\in Z_{q}^{*}$ and P, Q ∈G;
- Nondegeneracy: ∃P, Q ∈G, let $\hat{e}\left(P,Q\right)\neq 1$;
- Computability: ∀P, Q ∈G, $\hat{e}\left(P,Q\right)$ can be calculated efficiently.

### 3.2. ECDSA

The elliptic curve digital signature algorithm (ECDSA) is a simulation of the digital signature algorithm (DSA) based on the elliptic curve cryptography (ECC) algorithm. Let q, P be the public parameters as discussed above. ECDSA can be divided into the following three steps [33]:

- $ECKeyGen\left(\cdot \right)$: Select a random integer k as the private key, where $k\in \left[1,q-1\right]$. Compute the public key K=kP. The entity can obtain the key pair $\left(k,K\right)$.
- $ECSign\left(M\right)$: When receiving the message M, the entity first selects a random number $r\in \left[1,q-1\right]$, then generates rP. Set $\left(x,y\right)=rP$ and R=x mod q. Compute $h=SHA1\left(M\right)$, then generate $S=\left(h+k\;\cdot \;R\right)r^{-1}\;mod\;q$. Finally, the entity obtains the signature $\left(R,\;S\right)$. It is noted that if one of R or S equals 0, this algorithm should be rerun.
- $ECVerify\left(M,\left(R,S\right)\right)$: When the entity receives M and $\left(R,S\right)$, it first checks whether R and S are in the range $\left[1,q-1\right]$. If yes, the entity calculates $h^{\prime }=SHA1\left(M\right)$, $\left(x^{\prime },y^{\prime }\right)=h^{\prime }\cdot S^{-1}P+R\cdot S^{-1}K$ and $R^{\prime }=x^{\prime }\;mod\;q$. If $R^{\prime }=R$, the entity can accept the signature.

### 3.3. System Model

Referring to the overall architecture shown in Figure 1, the satellite network members for the SGIN in this article include the UE, regenerative LEO with gNB-DU (distributed unit), GS with gNB-CU (centralized unit), and NCC; the terrestrial network members include the UE, gNB, and 6GC. Due to the heterogeneous 6G environment and different user needs, we put forward two different scenarios of satellite network access: (1) An Unlinkable Authentication Scenario, which requires higher anonymity and unlinkability, based on a short group signature [23]; (2) a Batch Authentication Scenario, which includes massive lightweight UE, where their requirement for anonymity is relatively low. We designed different access authentication protocols for the two scenarios. The UE can select corresponding authentication modes based on their requirements. In addition, the proposed scheme provides roaming authentication methods for users who need to roam across domains.

**Figure 2:**
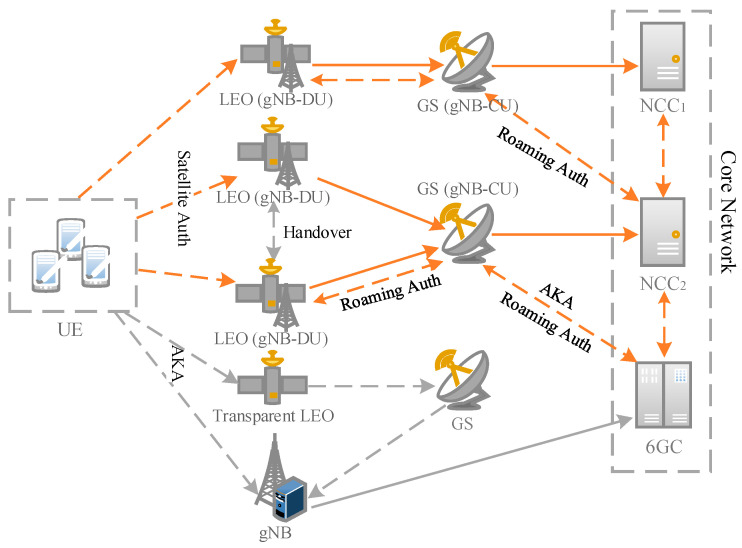
SGIN protocol model.

The protocol model of the SGIN is shown in Figure 2. The protocol involved in this paper is represented by orange lines. The dashed lines mean that the channel between two entities is insecure, and the solid lines are the contrary. The UE, connected to the satellite, can access the NCC through the two protocols designed in the following section. In addition, since regenerative satellites are equipped with gNBs, the UE can also access the 6G core network through regenerative satellites, transparent satellites, or gNBs, using the AKA protocol. When it is necessary to handover satellites due to their movement, the satellites plan the handover strategy independently. This process is transparent to the UE. When the UE needs to roam between networks of the same operator or roam across the networks of different operators, they have to use the protocols of the presented roaming authentication phase according to the situation. The protocol architecture aims to address the required access methods and performance for heterogeneous terminals.

### 3.4. Security Requirement

The proposed protocol has the following security assumptions:

- Assuming that the NCCs and 6GCs are trusted by UE, LEOs, and GSs. During the initialization phase, NCCs can send secret parameters for future use to the UE and LEOs over trusted channels (e.g., offline channels);
- Assuming that during satellite authentication, the channels between NCCs and GSs, GSs and LEOs, and NCCs and NCCs are trusted, it can be built using SSL or TLS. While using AKA, the satellite channels from UE to gNBs are untrusted, and the channels between gNBs and 6GCs are trusted;
- It is assumed that no trust relationship has been established between the UE and LEOs before access authentication;
- It is assumed that in an unlinkable authentication scenario, regenerative LEOs may track the message of the UE and try to reveal its real information;
- The proposed protocol also needs to meet the following security requirements:
- Forward and Backward Secrecy: Due to the changes in the geographical location and requirements of UE, the proposed scheme should ensure forward and backward security; that is, an attacker cannot obtain the current session key through the information of the previous session [34]. In addition, if the current session is compromised, the attacker cannot affect the security of the previous channel [35];
- Mutual Authentication: The proposed scheme should satisfy mutual authentication; that is, regenerative LEOs can detect and refuse access to an illegal UE. In addition, a UE can also know the legitimacy of access nodes in the system, to avoid potential malicious attacks;
- Key Establishment: The proposed scheme should ensure that the session keys negotiated by the protocol are only shared between the UE and regenerative LEOs;
- User Privacy: In the unlinkable authentication scenario, the UE requires unlinkability; that is, others cannot know whether the information comes from the same UE. In the batch authentication scenario and roaming authentication phase, the UE is linkable, but others will not be able to learn its identity information.

## 4. The Proposed Scheme

In this section, we give a detailed description of the scheme, which consists of four phases: an initialization phase, anonymous authentication phase, roaming authentication phase, and user revocation phase. Without losing generality, and in order to be more intuitive, we will only focus on a certain set of UE and the corresponding LEOs.

### 4.1. Initialization Phase

In the initialization phase, the satellite core network servers (i.e., NCCs) first input the security parameter λ∈N and generate the system master key $s\in Z_{q}^{*}$. Then, the NCC generates $\left(sk_{LEO},pk_{LEO}\right)$ for LEOs based on ECC. Moreover, the NCC outputs the public parameter $params=\langle q,G,G_{T},P,g,\hat{e},H_{1},H_{2}\rangle$, where G is the addictive cyclic group with the generator element $\left(P,g\right)$ and the order q, $G_{T}$ is the multiplicative cycle group with the same order q, $\hat{e}$ is the bilinear pairing $\hat{e}:G\times G\to G_{T}$, and $H_{1}:{\left\{0,1\right\}}^{*}\to Z_{q}^{*}$ and $H_{2}:{\left\{0,1\right\}}^{*}\times G\to Z_{q}^{*}$ are hash functions. At the same time, the 6GC completes the initial key configuration with the UE. The detailed access authentication protocols for terrestrial networks are beyond the scope of this paper.

After generating the public parameter, the NCC implements Algorithm 1 to generate the group public key gpk:〈g,h,u,v,ω〉 and group master secret key $gmsk:\langle \xi_{1},\xi_{2}\rangle$, in which gpk needs to be published in an open channel and gmsk needs to be kept secret by the NCC. For each $UE_{i}$ in the group, the NCC generates $gsk_{i}:\langle ID_{i},\varphi_{i},A_{i}\rangle$ and sends $gsk_{i}$ to the corresponding UE. The UE needs to use $gsk_{i}$ as its private key and not disclose it to anyone.
**Algorithm 1:** Initialization**Input**: a group of user identity, a number of users N**Output**: $\left(g,h,u,v,\omega ,\xi_{1},\xi_{2}\right)$, $\left(ID_{i},\;\varphi_{i},A_{i}\right)$1:Select random numbers u,v,h∈G2:Select random numbers $\xi_{1},\xi_{2},\;\gamma \in Z_{q}^{*}$ such that $\xi_{1}\cdot u=\xi_{2}\cdot v=h$3:Sets ω=γg4:**for** all $UE_{i}$ with identity $ID_{i}$
**do**5:      Select a random number $\varphi_{i}\in Z_{q}^{*}$6:      Set $A_{i}=\frac{1}{\gamma +\varphi_{i}}g$7:      Store the tuple $\left(ID_{i},\varphi_{i},A_{i}\right)$8:**end for**9:**return** $g,\;u,\;v,\;h,\;\omega ,\;\xi_{1},\xi_{2},\left(ID_{i},\varphi_{i},A_{i}\right)$

Additionally, the NCC generates the necessary parameters for batch authentication. The steps for batch authentication initialization are shown below:

1. NCC selects a random number $\varepsilon_{LEO}\in Z_{q}^{*}$ for each LEO, and generates a batch master key $BMK=s^{-1}P$ and batch public key $BPK_{LEO}=\varepsilon_{LEO}P$ for LEOs. The LEOs should keep BMK and $\varepsilon_{LEO}$ secret.
2. The NCC selects a random number $x_{i}\in Z_{q}^{*}$ for each $UE_{i}$. Then, the NCC calculates the batch authentication key $BK_{i}=x_{i}sP$, $RK_{i}=e\left(x_{i}P,P\right)$ for the UE. Therefore, the batch authentication key of $UE_{i}$ is $buk_{i}:\langle BK_{i},\;RK_{i}\rangle$, and the UE should keep $buk_{i}$ secret.

Finally, the NCC sends ($\varepsilon_{LEO},\;BMK,\;BPK_{LEO},\;sk_{LEO},pk_{LEO})$ to the LEOs over a secure channel (e.g., offline channel). Additionally, the NCC sends $\left(gsk_{i},gpk,buk_{i}\right)$ to the corresponding UE securely, and saves $ID_{i}$ and $gsk_{i}$ in a local user key list (UKL).

### 4.2. Anonymous Authentication Phase

In this section, we show two scenarios according to the different needs of the UE: an unlinkable authentication scenario and batch authentication scenario. Each UE gives all the key information required for the two scenarios during the initialization phase, so it can switch scenarios when needed, without reregistering.

#### 4.2.1. Unlinkable Authentication Scenario

**Figure 3:**
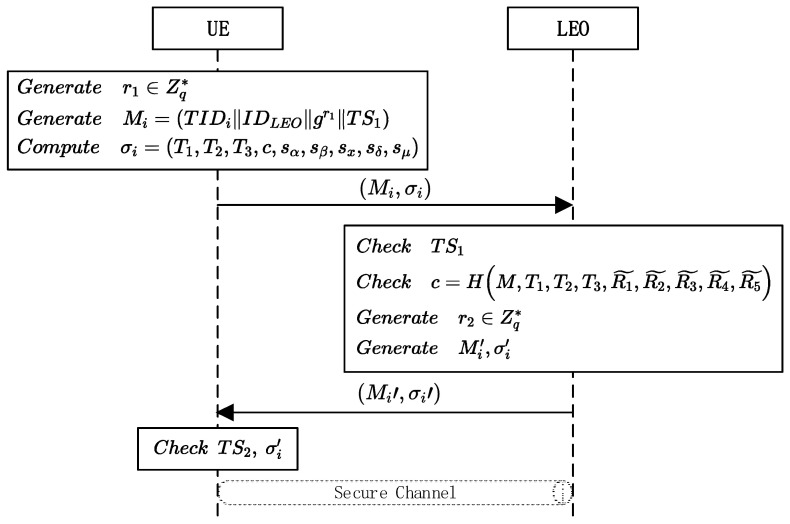
Authentication phase in the unlinkable authentication scenario.

In this scenario, we refer to Boneh’s [23] short group signature (SGS) algorithm, which is one of the most famous group signatures. The UE in SGS can randomly generate a temporary identity (TID) that is irrelevant to the real $ID_{i}$. Owing to the unlinkable anonymity of SGS, the UE can use different TIDs in different sessions, and no other entity knows that these TIDs belong to the same UE. The specific protocol process is shown in Figure 3, and its steps are as follows:

1. When the UE wants to access the NCC, the message $M_{i}=\left(TID_{i}\left|\left|ID_{LEO}\right|\right|g^{r_{1}}||TS_{1}\right)$ needs to be constructed, where $r_{1}\in Z_{q}^{*}$ is a random number, $g^{r_{1}}$ is part of the session key, and $TS_{1}$ is a timestamp that can resist reply attacks. Taking $M_{i}$, gpk, and $gsk_{i}$ as input, the UE implements Algorithm 2 and obtains the signature $\sigma_{i}=\left(T_{1},T_{2},T_{3},c,s_{\alpha },s_{\beta },s_{x},s_{\delta },s_{\mu }\right)$, where $\hat{e}\left(A_{i},g\right)$, $\hat{e}\left(h,g\right)$ and $\hat{e}\left(h,\omega \right)$ can be calculated and stored in advance. When the UE needs to revote its secret keys, it needs to recalculate $\hat{e}\left(\hat{A_{i}},g\right)$ and $\hat{e}\left(h,\;\hat{\omega }\right)$. After completing the construction of plaintext and signature, the UE sends $\left(M_{i},\sigma_{i}\right)$ to an appropriate LEO.
2. When receiving s request from the UE, the LEO first authenticates the timestamp $TS_{1}$ in the message. The LEO generates the current timestamp $TS_{1}^{\prime }$ and verifies $TS_{1}^{\prime }-TS_{1}<\Delta T$, where ΔT is adjusted according to different network conditions. If it does not meet the conditions, the LEO returns an error message. Otherwise, the LEO validates the accuracy of the signature using Algorithm 3. If the verification passes, the LEO selects the random number $r_{2}\in Z_{q}^{*}$ and calculates the session key $SK={\left(g^{r_{1}}\right)}^{r_{2}}$. If the verification fails, an error message is returned. The LEO signs the message $M_{i}^{\prime }=\left(TID_{i}\left|\left|ID_{LEO}\right|\right|g^{r_{2}}||TS_{2}\right)$ using $ECSign\left(M_{i}^{\prime }\right)$ to obtain the signature $\sigma_{i}^{\prime }$. Then, the LEO sends the message $\left(M_{i}^{\prime },\sigma_{i}^{\prime }\right)$ to the UE.
3. After receiving the message, the UE first verifies the validity of the timestamp; that is, whether the timestamp satisfies $TS_{2}^{\prime }-TS_{2}<\Delta T$. If it is valid, the message will be verified by the ECDSA. If verified, the UE calculates the session key $SK={\left(g^{r_{2}}\right)}^{r_{1}}$. The key negotiation between the two sides is complete.

**Algorithm 2:** Generating Signature**Input:** $M_{i},gpk,gsk_{i}$**Output:** $\sigma_{i}$1:Select random numbers $\alpha ,\beta \in Z_{q}^{*}$2:Set $T_{1}=\alpha u$, $T_{2}=\beta v$, $T_{3}=A_{i}+\left(\alpha +\beta \right)h$3:Set $\delta =\alpha \varphi_{i}$ and $\mu =\beta \varphi_{i}$4:Select random numbers $r_{\alpha },\;r_{\beta },r_{x},r_{\delta },r_{\mu }\in Z_{q}^{*}$5:Set $R_{1}=r_{\alpha }u,$ $R_{2}=r_{\beta }v,$ $R_{4}=r_{x}T_{1}-r_{\delta }u,$ $R_{5}=r_{x}T_{2}-r_{\mu }v,\;$ $R_{3}=\hat{e}{\left(T_{3},g\right)}^{r_{x}}\hat{e}\left(h,\left(-r_{\alpha }-r_{\beta }\right)\omega +\left(-r_{\delta }-r_{\mu }\right)g\right)$ $=\hat{e}{\left(A_{i},g\right)}^{r_{x}}\;\hat{e}{\left(h,g\right)}^{r_{x}\left(\alpha +\beta \right)}\;\hat{e}{\left(h,g\right)}^{-r_{\delta }-r_{\mu }}\;\hat{e}{\left(h,\omega \right)}^{-r_{\alpha }-r_{\beta }}\;$6:Set $c=H\left(M,T_{1},T_{2},T_{3},R_{1},R_{2},R_{3},R_{4},R_{5}\right)\in Z_{q}^{*}$7:Set $s_{\alpha }=r_{\alpha }+c$, $s_{\beta }=r_{\beta }+c$, $s_{x}=r_{x}+cx$, $s_{\delta }=r_{\delta }+c$, $s_{\mu }=r_{\mu }+c$8:**return** $\sigma_{i}=\left(T_{1},T_{2},T_{3},c,s_{\alpha },s_{\beta },s_{x},s_{\delta },s_{\mu }\right)$

**Algorithm 3:** Verifying Message**Input:** $M_{i},\sigma ,gpk$**Output:** ∅1:Set $\tilde{R_{1}}=-cT_{1}+s_{\alpha }u,$ $\tilde{R_{2}}=-cT_{2}+s_{\beta }v,$ $\tilde{R_{4}}=s_{x}T_{1}-s_{\delta }u,$ $\tilde{R_{5}}=s_{x}T_{2}-s_{\mu }v,$ $\tilde{R_{3}}=\hat{e}\left(s_{x}T_{3},g\right)\hat{e}\left(cT_{3},\omega \right)\hat{e}{\left(h,\omega \right)}^{-s_{\alpha }-s_{\beta }}\hat{e}{\left(h,g\right)}^{-s_{\delta }-s_{\mu }}\hat{e}{\left(g,g\right)}^{-c}$2:**If** $c=H(M_{i},T_{1},T_{2},T_{3},\tilde{R_{1}},\tilde{R_{2}},\tilde{R_{3}},\tilde{R_{4}},\tilde{R_{5}})$ **then**3:        The signature $\sigma_{i}$ is valid4:
**else**
5:        Reject the signature6:
**end if**


#### 4.2.2. Batch Authentication Scenario

**Figure 4:**
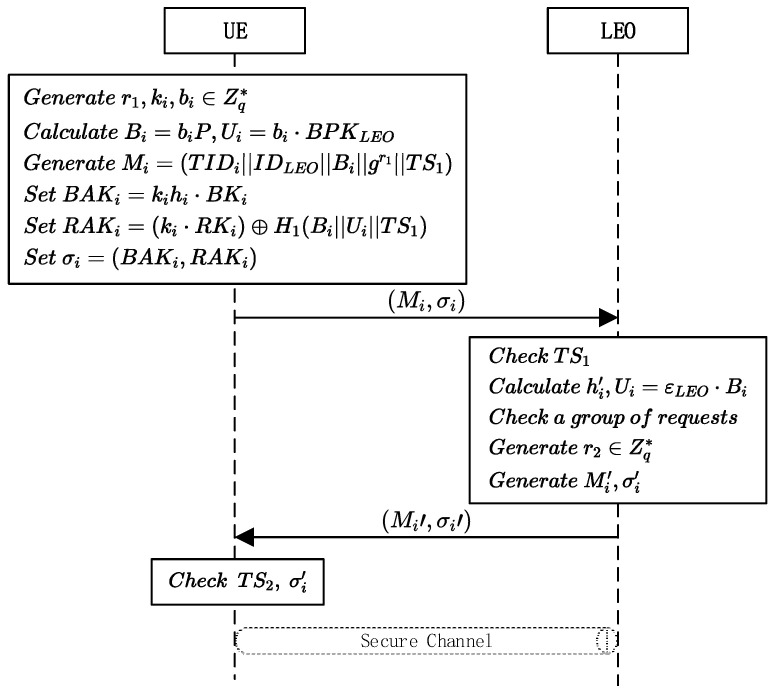
Authentication phase in the batch authentication scenario.

The traditional short group signature scheme uses a batch group signature (BGS) to complete batch authentication. However, due to the insufficient computing power of some devices in 6G heterogeneous networks and the massive terminals, we designed a novel batch authentication protocol to meet the needs of these terminals. In this scenario, users can generate a TID, but it is traceable. A set of UE send their batch authentication message to the LEO, which authenticates all parameters uniformly. If the first batch authentication is successful, the LEO authenticates them and continues the authentication process. If the first authentication fails, a rebatch is required. The specific protocol process is shown in Figure 4, and the detailed steps are as follows:

1. The UE selects random numbers $r_{1},k_{i},b_{i}\in Z_{q}^{*}$, then calculates $B_{i}=b_{i}P$, $U_{i}=b_{i}\cdot BPK_{LEO}$. The UE sets the access message as $M_{i}=\left(TID_{i}\left|\left|ID_{LEO}\right|\right|B_{i}\left|\left|g^{r_{1}}\right|\right|TS_{1}\right)$, where $TS_{1}$ is a timestamp. Then, UE calculates the hash value $h_{i}=H_{1}\left(M_{i}\right)$ of $M_{i}$, and sets the batch authentication key $BAK_{i}=k_{i}h_{i}\cdot BK_{i}$ and $RAK_{i}=\left(k_{i}\cdot RK_{i}\right)⊕H_{1}\left(B_{i}\left|\left|U_{i}\;\right|\right|TS_{1}\right)$. Then, the UE receives the signature of batch authentication $\sigma_{i}=\left(BAK_{i},RAK_{i}\right)$. After signing the message, the UE sends $\left(M_{i},\sigma_{i}\right)$ to the target LEO;
2. When the LEO receives $\left(M_{i},\sigma_{i}\right)$ from the UE, it first checks the validity of the timestamp $TS_{1}$. If $TS_{1}$ is legal, the LEO sets $h_{i}’=H_{1}\left(M_{i}\right)$, $U_{i}=\varepsilon_{LEO}\cdot B_{i}$ and $H_{1}\left(B_{i}\left|\left|U_{i}\right|\right|TS_{1}\right)$, then calculates the following equation:
  (1) $$e(\overset{n}{\underset{i=1}{\sum }}BAK_{i},BMK)=\prod_{i=1}^{n}h_{i}\left(RAK_{i}⊕H_{1}\left(B_{i}\left|\left|U_{i}\right|\right|TS_{1}\right)\right)$$
  If Equation (1) is true, n UE in the batch authentication group is valid. Otherwise, it means that there are invalid messages in this group. Batch authentication has the advantage of reducing the computational overheads, but once an invalid request occurs in a batch, the authentication will fail. When a malicious attacker continuously sends invalid information to implement DoS attacks, users may be unable to complete authentication for a long time. Therefore, a rebatch is required to protect the UE’s QoS. The algorithm of “divide-and-conquer” (BVDC) [25] can be used. The LEO can use dichotomous validation for a batch authenticated UE, to find the UE that failed validation and return error messages. Although the rebatch may bring computation overheads, it is helpful for improving the overall system efficiency and increasing the verification success rate.
3. For UE that passes the authentication, the LEO constructs $M_{i}^{\prime }=\left(TID_{i}\left|\left|ID_{LEO}\right|\right|g^{r_{2}}||TS_{2}\right)$, where $TS_{2}$ is a timestamp, $g^{r_{2}}$ is a session key parameter generated by LEO, and $r_{2}\in Z_{q}^{*}$ is a number randomly generated. The LEO uses $ECSign\left(M_{i}^{\prime }\right)$ to generate the signature $\sigma_{i}^{\prime }$ and send $\left(M_{i}^{\prime },\sigma_{i}^{\prime }\right)$ to the UE.
4. After receiving the message, the UE first verifies the validity of the timestamp. The message is then verified by $ECVerify\left(M_{i}^{\prime },\;\sigma_{i}^{\prime }\right)$. If the verification is successful, the UE calculates the session key $SK={\left(g^{r_{2}}\right)}^{r_{1}}$, and the LEO calculates the session key $SK={\left(g^{r_{1}}\right)}^{r_{2}}$. The key negotiation between the two sides is complete.

### 4.3. Roaming Authentication Phase

When the UE needs to roam across the network, due to changes in geographical location or network conditions, the roaming authentication phase can be completed. The proposed scheme designs a lightweight roaming authentication protocol to meet the needs of UE. For UE that need to change core network, they must be re-authenticated and negotiate a new session key. To reduce the overheads of roaming authentication, the UE should perform pre-negotiation after the initial authentication or the last roaming.

**Figure 5:**
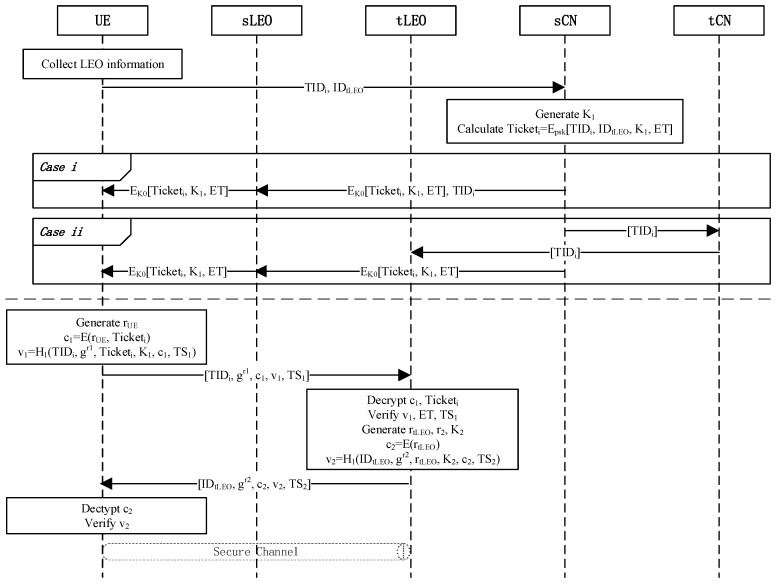
Pre-negotiation and roaming authentication phase in SGIN.

**Pre-negotiation Phase**: The UE first collects optional satellite information, and then sends its $TID_{i}$ and $ID_{tLEO}$ to the source core network, namely sCN (i.e., NCCs or 6GCs) through the sLEO, to request roaming authentication tokens. After receiving the UE’s request information, the sCN selects the generation key $K_{1}$. Then, sCN calculates $Ticket_{i}=SENC_{KDF\left(psk\right)}\left(TID_{i}\left|\left|ID_{tLEO}\right|\right|K_{1}||ET\right)$, where $SENC\left(\cdot \right)$ is a symmetric encryption function, $KDF\left(\cdot \right)$ is a key derivation function, psk is a pre-shared key maintained between the CNs and LEOs, and ET is the expiration time of the token. A secure channel is established between the UE and sLEO during the authentication phase, and secure channels exist between core networks. The sCN encrypts $\left(Ticket_{i},K_{1},ET\right)$ using the key $K_{0}$ stored between sCN and UE, then returns the message to the UE. When $Ticket_{i}$ expires or the UE discovers new suitable LEOs, the UE needs to apply to the sLEO for new tokens. The LEO updates the token issued to the UE when the LEO or CN evaluates that it is necessary to change the psk. At the same time, if the tLEO does not belong to the sCN as *Case ii*, the sCN sends the $TID_{i}$ list to the tCN through the channel, then tCN sends $TID_{i}$ to tLEO. Otherwise, sCN sends the $TID_{i}$ list directly to tLEO as *Case i*. The specific negotiation process is shown in the upper part of Figure 5.

**Roaming Authentication Phase**: When the UE needs to roam, the process is as shown in the lower part of Figure 5. The following steps need to be completed:

1. The UE first generates a random number $r_{UE}$, then uses the public key $pk_{tLEO}$ of the tLEO to encrypt $c_{1}=AENC_{pk_{tLEO}}\left(r_{UE},Ticket_{i}\right)$, where $AENC\left(\cdot \right)$ is an asymmetric encryption algorithm based on ECC. Then, the UE selects a random number $r_{1}\in Z_{q}^{*}$ and obtains a timestamp $TS_{1}$, sets $g^{r_{1}}$, and generates $v_{1}=H_{1}\left(TID_{i}||g^{r_{1}}\left|\left|Ticket_{i}\right|\right|K_{1}\left|\left|c_{1}\right|\right|TS_{1}\right)$. Finally, the UE sends message $M_{1}=\left(TID_{i}\left|\left|g^{r_{1}}\left|\left|c_{1}\right|\right|v_{1}\right|\right|TS_{1}\right)$ to the tLEO;
2. Upon receiving the message, the tLEO first checks the timestamp then decrypts $c_{1}$ using its private key $sk_{tLEO}$ in order to obtain $r_{UE}$ and $Ticket_{i}$. The tLEO decrypts $Ticket_{i}$ using $KDF\left(psk\right)$ and checks the ID and ET in the token. If it does meet the conditions, the tLEO generates $v_{1}^{\prime }$ to verify whether it is equal to $v_{1}$. If verified, tLEO generates a random number $r_{tLEO}$ and generates $K_{2}=KDF\left(K_{1}||r_{UE}\right)$. Then, the tLEO calculates $c_{2}=SENC_{K_{2}}\left(r_{tLEO}\right)$, where $SENC\left(m\right)$ is a symmetric encryption algorithm. The tLEO selects a random number $r_{2}\in Z_{q}^{*}$ and sets $g^{r_{2}}$. Finally, the tLEO calculates $v_{2}=H_{1}\left(ID_{tLEO}||g^{r_{2}}\left|\left|r_{tLEO}\right|\right|K_{2}\left|\left|c_{2}\right|\right|TS_{2}\right)$ and sends the message $M_{2}=\left(ID_{tLEO}\left|\left|g^{r_{2}}\left|\left|c_{2}\right|\right|v_{2}\right|\right|TS_{2}\right)$ to the UE, where $TS_{2}$ is a timestamp;
3. While receiving the message, UE first checks the timestamp. If checks, UE calculates $K_{2}=KDF\left(K_{1}||r_{UE}\right)$. Then UE decrypts $c_{2}$ and gets $r_{tLEO}$. UE calculates $v_{2}^{\prime }$ and checks whether $v_{2}^{\prime }=v_{2}$. If it does meet, the new conversation between UE and the tLEO is established. UE and the tLEO get their new session keys $SK={\left(g^{r_{2}}\right)}^{r_{1}}$ and $SK={\left(g^{r_{1}}\right)}^{r_{2}}$.

### 4.4. User Revocation Phase

In the case that the UE needs to quit the group or the system needs to revoke the illegal UE authentication in the unlinkable authentication, the algorithm in the user revocation phase is needed. For an illegal UE, the NCC has the right to disclose their real ID and other information through signatures they send out. The private key of the illegal UE can be calculated through the group master key $gmsk:\langle \xi_{1},\xi_{2}\rangle$ and $\left(T_{1},T_{2},T_{3}\right)$ in the signature. The NCC can find the real $ID_{i}$ of the UE by comparing with the user information in UKL. For an illegal UE that requests to quit the group, the NCC performs the operations described above. After that, the NCC creates a revocation list (RL) that contains the key $\left(\varphi_{j},A_{j},RK_{j}\right)$ of the $UE_{j}$ to be revoked. The NCC sends the RL to each LEO. The LEOs save the RL and periodically broadcast the latest RL’, which includes $\left(\varphi_{j},A_{j}\right)$.

A UE that receives the RL’ updates its private key according to Algorithm 4, where m is the total number of tuples in RL’. Unrevoked UE must run this algorithm until all UEs in RL’ are revoked. After completing the above steps, the unrevoked UE needs to update the pre-stored parameters $\hat{e}\left(\hat{A_{i}},g\right)$ and $\hat{e}\left(h,\;\hat{\omega }\right)$. In addition, for offline UE, they need to request the latest RL’ when they are online. Therefore, the revoked $UE_{j}$ cannot obtain a new $gsk_{j}$. The authentication will fail when the $UE_{j}$ participates in group signature authentication again. When participating in batch authentication, $UE_{j}$ will also be detected by the LEO, thus prohibiting its access to the network.
**Algorithm 4:** Unrevoked User Update Parameters**Input:** $gsk_{i},RL\prime =\{x_{j},A_{j}|1\leq j\leq m\}$**Output:** $\left(\varphi_{i},\hat{A_{i}}\right)$1:Update g as $\hat{g}=A_{j}^{*}=\frac{1}{\varphi_{j}+\gamma }g$2:Update $\hat{\omega }=g-\varphi_{j}\cdot A_{j}=\gamma \cdot \hat{g}$3:Update the secret key as $\hat{A_{i}}=\frac{1}{\varphi_{i}-\varphi_{j}}\cdot A_{j}-\frac{1}{\varphi_{i}-\varphi_{j}}\cdot A_{i}=\frac{1}{\varphi_{i}+\gamma }\cdot \hat{g}$4:**return** $\left(\varphi_{i},\hat{A_{i}}\right)$

The user revocation process can be carried out offline, which reduces the burden on the UE and prevents delays caused by the LEO checking the RL operation during authentication. The performance analysis of Yang et al. [30] showed that the revocation mechanism of SGS is effective. For the protocol that is unlinkable, this phase allows the UE to exit the group, better managing the network. Although user revocation brings additional computational overheads, it is acceptable and necessary.

## 5. Security Verification

In this section, we use the ProVerif tool to conduct formal verification of the protocol. Then, we complete an informal security analysis.

### 5.1. Formal Analysis Using ProVerif

We used the ProVerif tool to formalize the proposed protocol in two parts. ProVerif is an automated protocol verification tool that emulates protocols and validates secure protocols against known active and passive attacks. It can handle various encryption primitives, such as key exchange schemes, hash functions, asymmetric encryption, and symmetric encryption. Since the proposed scheme is based on bilinear mapping, we used equations in ProVerif to add specific rules to analyze the protocol more accurately.

**Figure 6:**
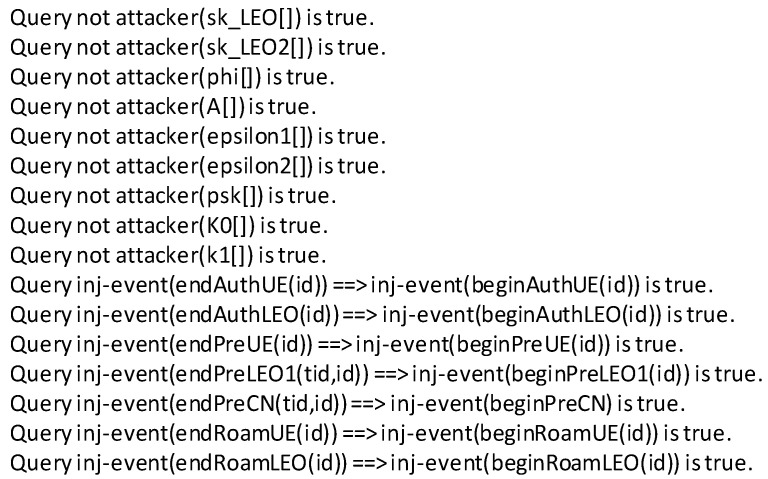
Results from the implementations of the unlinkable authentication scenario using ProVerif.

**Figure 7:**
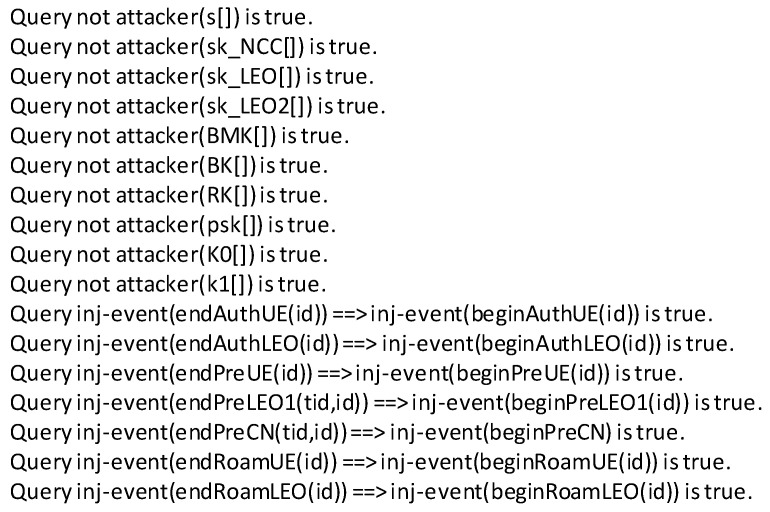
Results from the implementations of the batch authentication scenario using ProVerif.

The ProVerif code of the proposed scheme consists of two parts: unlinkable authentication verification and batch authentication verification. The roaming authentication phase is included in each part. All verification results are shown in Figure 6 and Figure 7. Specially, *sk_LEO* and *sk_LEO2* indicate secret keys for different LEOs; and *psk*, *K0*, and *k1* indicate the keys used in the roaming authentication phase described in Section 4.3. For unlinkable authentication verification, (*phi*, *A*) and (*epsilon1*, *epsilon2*) indicate the UE’s group secret keys and NCC’s group master secret keys in Section 4.1. For batch authentication verification, *s* and *sk_NCC* indicate the secret keys of the NCC, and (*BMK*, *BK*, *RK*) indicate the keys used in batch authentication in Section 4.1. There are injective correspondences between the participants in each step of authentication, pre-negotiation, and roaming authentication. The figures show that the two parts of the proposed scheme are reliable, and the individual keys and the negotiated session keys are secure.

### 5.2. Informal Security Analysis

In this section, we analyze important security characteristics of the proposed scheme. In the first four sections, we analyze the security characteristics of the proposed scheme. In the subsequent four sections, we analyze attacks that the proposed scheme can defend against.

1. 1. Mutual Authentication and Key Establishment

In a SGIN, authentication passes through at least two interactions. In the first step, the UE sends a message to the LEO, and the UE is authenticated by the LEO. In the second step, the LEO returns the signature of the ECSDA to the UE, and the identity of the LEO is proven by the UE. This completes the process of mutual authentication between the UE and LEO. The keys of both the UE and LEO are issued by the trusted NCC during the initialization phase, and the authenticator holds the public keys of the target nodes, such as gpk and $pk_{LEO}$. Therefore, the fake group members or LEOs cannot be authenticated by the legitimate node.

2. Session Key Security

In each phase of the proposed scheme, no matter in which scenario, the session key is calculated according to the Diffie–Hellman problem through two random numbers generated by the two entities (i.e., UE and LEO). Calculating the session key without knowing the two generators involves solving the discrete logarithm problem on an elliptic curve. At present, it is computationally infeasible to solve the discrete logarithm problem in polynomial time [36].

3. Forward/Backward Security

New session keys $SK={\left(g^{r_{1}}\right)}^{r_{2}}$ and $SK={\left(g^{r_{2}}\right)}^{r_{1}}$ are negotiated in both authentication and roaming authentication phases of the proposed protocol. There is no computable correlation between the new session key and the session key of other sessions. No entity other than the UE and LEO can calculate the new session key.

4. Privacy and Untraceability

In SGIN unlinkable authentication scenario, it is difficult for an attacker to know the real identity of a UE through the signature. Unless the attacker can obtain the UE’s private key and the UKL stored in the NCC, it cannot reveal the UE’s identity. Or in another case, the attacker obtains gmsk. But these are extremely difficult to do for the attacker. From another perspective, anonymity in the unlinkable authentication scenario is conditional. NCC has the right to recover the UE’s private key $A_{i}$ of the signature through gmsk, so as to obtain the real identity of the UE. In addition, the anonymity is untraceable, and the UE can use different TID in different sessions, and the attacker cannot associate two different sessions of the same UE through signatures.

In the batch authentication scenario, the UE is traceable due to the existence of RK. However, it is difficult for the attacker to crack the real ID of the UE using the batch authentication key RAK. In addition, the UE in the unlinkable authentication scenario does not send information correlating to batch authentication. Even if the UE is changed, the attacker cannot associate the UE in batch authentication scenario with the UE in the unlinkable authentication scenario.

5. Resistance to Replay Attacks

In each scenario, the entity sends a message M that contains a timestamp TS. The integrity of the timestamp is protected by a signature, so it is difficult for an attacker to tamper with the timestamp. The other entity verifies the timestamp $TS^{\prime }-TS<\Delta T$, where ΔT is the interval that matches the current network condition. Therefore, the authentication party can confirm the freshness of the message and distinguish whether it is under replay attack.

6. Resistance of Impersonation Attacks

Suppose an attacker tries to imitate a legitimate UE, it must have the UE’s group private key $gsk_{i}$, batch private key $buk_{i}$, or roaming parameters in order to generate a valid signature. However, these private keys are only held by the UE and NCC. It is difficult for the attacker to obtain both private keys. The LEO’s private key is only held by the LEO and NCC. If the attacker uses the wrong private key signature, the UE will verify the signature and the validation will fail. In the roaming authentication phase, the attacker needs to obtain the psk, modify the information in the token, and send the token to the UE through the established secure channel to complete the attack. This is also difficult for the attacker.

7. Resistance to Man-in-the-Middle Attacks

An attacker attempting a man-in-the-middle attack attempts to intercept the communication between the UE and LEO and imitate the other party in the conversation. However, due to the resistance of the protocol to impersonation attacks, it is difficult for the attacker to successfully achieve this goal, and they therefore cannot complete man-in-the-middle attacks.

8. Resistance to Dos Attacks

The traditional batch authentication protocol does not support a reauthentication algorithm. Therefore, when an attacker launches a DoS attack, the UE of the whole group cannot perform batch authentication. Thus, the LEO cannot know the specific UE who implemented the DoS attack. The batch authentication scenario of the proposed scheme supports the rebatch process. A legitimate UE can pass the authentication without re-authentication, and the LEO can also find the illegal UE that launched attacks. When the number of illegal operations performed by a UE reaches the threshold, the NCC can revoke them.

## 6. Performance Analysis

In this section, the computational complexity and communication overheads of the proposed scheme are analyzed.

### 6.1. Computational Complexity Analysis

#### 6.1.1. Computational Complexity in the Unlinkable Authentication Scenario

In the unlinkable authentication scenario, we compared the protocol with the work of Feng et al. [37], Alamer [24], and Wasef et al. [12]. The operations involved in these protocols are shown in Table 2, where G symbolizes the addictive cyclic group of prime order q, and $G_{T}$ symbolizes multiplicative addictive cyclic group of the same order. Moreover, $g_{1}$, $g_{2}$, $g_{T_{1}}$, and $g_{T_{2}}$ are generators, where $g_{1},g_{2}\in G$ and $g_{T_{1}},g_{T_{2}}\in G_{T}$. Parameters a and b are random numbers in $Z_{q}^{*}$.

In order to analyze the performance of each architecture, we designed an experiment based on the OpenSSL library, GMP library, and PBC library and tested on an Ubuntu 20.04.3 64-bit 4 GB virtual machine with 16 GB memory and a 3.20 GHz 8 core AMD CPU hardware configuration. We tested each operation 10,000 times and calculated their average value. The specific cost of each operation is shown in Table 2. As can be seen from Table 2, $T_{Pairing}$, $T_{Mul_{1}}$, $T_{Exp}$, $T_{Hash_{2}}$, and $T_{Hash_{3}}$ were more time-consuming for all operations. Therefore, in the following analysis of each scheme, attention was only paid to the impact of these five operations on the performance.

A comparison of related works in the unlinkable authentication scenario is shown in Table 3. It should be noted that although a group signature is used, the application scenarios and architectures of these works are different. We only compared the steps of the individual signature and verification. The initialization and registration steps take place before the entire system starts, and their overheads can be excluded from the authentication overhead.

In order to protect the unlinkability, the proposed protocol cannot directly use the UE’s private key when the UE sends signatures to the LEO. Otherwise, other entities could verify the signature through the UE’s public key and easily trace it. Since entities can know the identity of the satellite through analysis of the orbit of the satellite, there is no need to protect the privacy of the satellite node. Therefore, the LEO can use a signature algorithm with lower cost (i.e., ECDSA) when it sends signatures to the UE. The computational cost of the ECSDA is only the $1T_{Mul_{1}}$ operation for signature and $2T_{Mul_{1}}$ operations for verification, whose cost is small compared with other signature algorithms. As shown in Table 3, one-way authentication in the proposed scheme requires $19T_{Mul_{1}}$, $2T_{Pairing}$, and $7T_{Exp}$. It takes about 20 ms for the signature and verification in the unlinkable authentication scenario, which is less time than the other schemes.

#### 6.1.2. Computational Complexity in the Batch Authentication Scenario

Considering the large number of UEs in some scenarios, it is necessary to reduce the amount of authentication data and the processing time of authentication requests [16]. The batch authentication scenario in the proposed scheme can provide efficient services for these UE and lighten the LEOs’ burden. We evaluated the cost of the first batch authentication and the rebatch authentication. We also estimated the computational complexity of the rebatch authentication.

**Figure 8:**
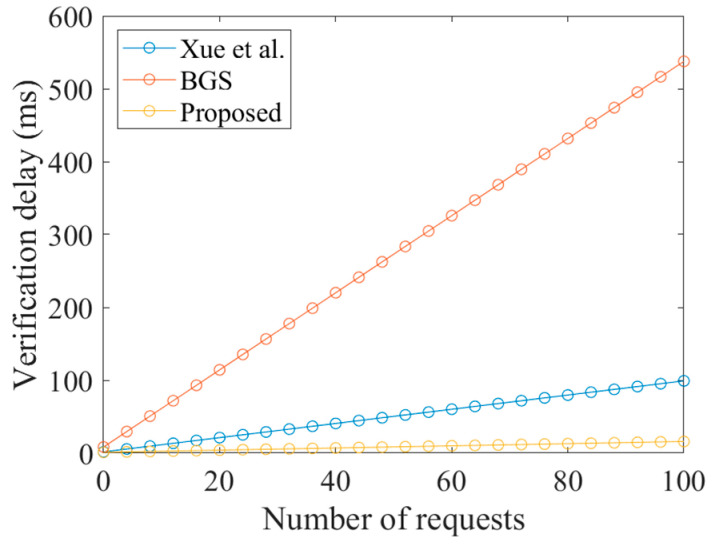
Computational cost comparison of batch authentication in the verification phase. Xue et al. [11], BGS [12].

In the evaluation of first batch verification, we compared the proposed scheme with the related works of Xue et al. [11] and Wasef et al. [12]. In order to contrast with the unlinkable authentication scenario, we compared with the work of Wasef et al. [12], based on a batch group signature (BGS). The cost of signing and verifying are shown in Table 4. Although the computational complexity of the signature of the proposed scheme was higher than that of Xue et al.’s work, Xue et al. [11] used a UE private key to complete the signature, and the LEO needs to use the UE’s public key to verify the signature. This is detrimental to the privacy protection of the UE, as attackers can easily trace the UE through its public key. Therefore, based on security and privacy considerations, our scheme is more suitable for the proposed SGIN scenario. Additionally, Figure 8 shows that the verification cost of the three schemes varies with the increase in the number of authentication requests. It can be seen that the cost of the proposed scheme is significantly less than that required for BGS and the work of Xue et al. [11].

In terms of rebatch authentication, it is assumed that there is at most a 1% vulnerable UE in a group containing 1000 UE for batch authentication, then the maximum number of UEs breached in this group is $N_{ckd}=N_{all}\times 1\%=1000\times 1\%=10$. Given that the number of request messages in a batch is $N_{req}$, the probability that $N_{req}$ requests contain exactly i invalid requests can be expressed using the hypergeometric distribution as

(2) $$Pr\{X=i\}=\frac{\left(\begin{array}{c} N_{all}-N_{ckd}\\ N_{req}-i \end{array}\right)\left(\begin{array}{c} N_{ckd}\\ i \end{array}\right)}{\left(\begin{array}{c} N_{all}\\ N_{req} \end{array}\right)}$$

Assuming that event A means rebatch authentication is required to successfully verify valid requests, the probability of event A can be expressed as

(3) $$\begin{array}{cc} & Pr\left\{A\right\}=Pr\{i=1\}+\cdots +Pr\{i=10\}\\ = & \frac{\left(\begin{array}{c} N_{all}-N_{ckd}\\ N_{req}-1 \end{array}\right)\left(\begin{array}{c} N_{ckd}\\ 1 \end{array}\right)}{\left(\begin{array}{c} N_{all}\\ N_{req} \end{array}\right)}+\cdots +\frac{\left(\begin{array}{c} N_{all}-N_{ckd}\\ N_{req}-10 \end{array}\right)\left(\begin{array}{c} N_{ckd}\\ 10 \end{array}\right)}{\left(\begin{array}{c} N_{all}\\ N_{req} \end{array}\right)}\\ = & \frac{\sum_{i=1}^{10}\left(\begin{array}{c} N_{all}-N_{ckd}\\ N_{req}-i \end{array}\right)\left(\begin{array}{c} N_{ckd}\\ i \end{array}\right)}{\left(\begin{array}{c} N_{all}\\ N_{req} \end{array}\right)} \end{array}$$

According to Equation (3), when there are only one or two invalid requests (i=1 or i=2) in a batch, the probability of rebatch authentication is extremely small. However, in the case of DoS attacks by malicious UEs, rebatch authentication can protect a legitimate UE from authentication failure for a long time.

The proposed scheme in the first authentication needs $nT_{Exp}$ and $1T_{Pairing}$ operation. The LEO calculates $h_{i}\left(RAK_{i}⊕H_{1}\left(B_{i}\left|\left|U_{i}\right|\right|TS_{1}\right)\right)$ for each UE in the first authentication phase, thus only the $T_{Pairing}$ operation is required. Therefore, the computational cost of the worst and average cases of rebatch authentication is analyzed below:

1. Worst case: According to the rebatch algorithm proposed by Huang et al. [25], we assume that the worst case is where invalid requests are always in the detected batch. Assuming there are n requests in a batch, it takes ${log}_{2}n$ times the calculation in the worst case. Therefore, the worst-case total batch validation time for a valid request is
  (4) $$T_{wst}=T_{fst}+2\times {log}_{2}n\times T_{reb}$$
  where $T_{wst}$ is the time required for the worst case, $T_{fst}$ is the time required for the first batch verification, and $T_{reb}$ is the time required for completion of the rebatch algorithm.
2. Average case: Calculating the total validation cost in all cases divided by the number of possible cases gives the validation time required for the average case:
  (5) $$T_{ave}=T_{fst}+\frac{1}{{log}_{2}n+1}\sum_{i=2}^{{log}_{2}n}T_{reb}$$

**Figure 9:**
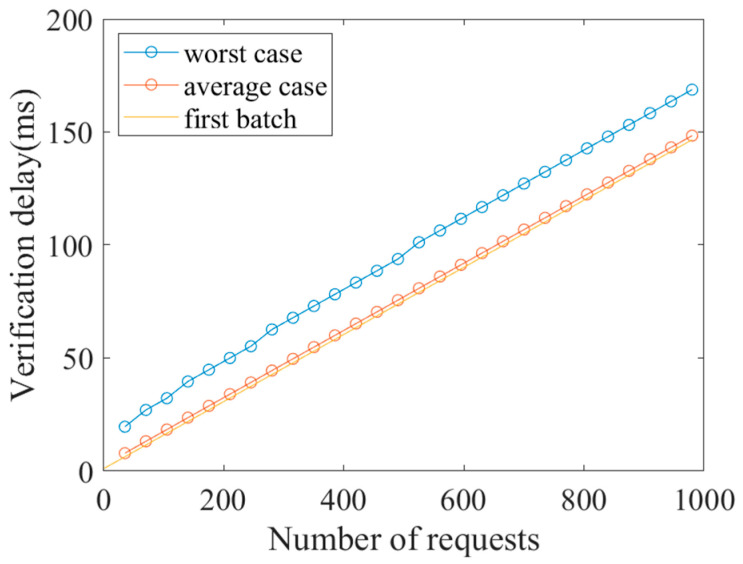
Case comparison for rebatch authentication.

Figure 9 shows the total validation complexity for the worst case, average case, and first batch authentication with 0 to 1000 UE requests. As can be seen from the figure, although the complexity of rebatch authentication in the worst case is relatively high, the cost of rebatch authentication in the average case is close to the complexity of the initial batch authentication, and the computational complexity does not increase rapidly with the increase of the number of requests. Considering the possibility of DoS attacks leading to large-scale UE authentication failure, rebatch authentication is necessary.

#### 6.1.3. Computational Complexity in Roaming Authentication

In the process of roaming authentication, we use symmetric encryption to ensure the efficiency of the scheme. The AES CBC algorithm was tested using OpenSSL library in the same environment. We tested the symmetric encryption and decryption algorithms 10,000 times and took their average values: the encryption algorithm costs 0.000112 ms, and the decryption algorithm costs 0.000110 ms. Assuming that the times required for symmetric encryption of the AES algorithm are $T_{senc}$ and $T_{sdec}$, and compared with the overheads in Table 2, they are negligible. In order to be consistent with the anonymous authentication phase, roaming authentication uses the same session key generation method. The proposed roaming authentication protocol involves $2T_{Exp}$ operations to negotiate the session key. It was assumed that the time required for the encryption algorithm in asymmetric encryption is $T_{aenc}$ and the time required for the decryption algorithm is $T_{adec}$. After 10,000 calculations using the OpenSSL library, the average value was obtained: $T_{aenc}$ = 0.015079 ms, $T_{adec}$ = 0.028344 ms. Then the computational cost required for the UE to send a message to LEO was $T_{Exp}$ = 0.148430 ms. The computational cost for the LEO to send a message to the UE was $T_{aenc}+T_{Exp}$ = 0.176774 ms. It was shown that the total roaming authentication calculation cost is far less than the cost of re-authentication.

### 6.2. Communication Overhead Analysis

According to the simulation results, the size of elements $L_{G}$ and $L_{Z}$ in G and $Z_{q}^{*}$ are both 16 bytes. Assuming that the identity length $L_{ID}$ and time message length $L_{T}$ are both 12 bytes. The hash function length $L_{H}$ is 32 bytes.

**Figure 10:**
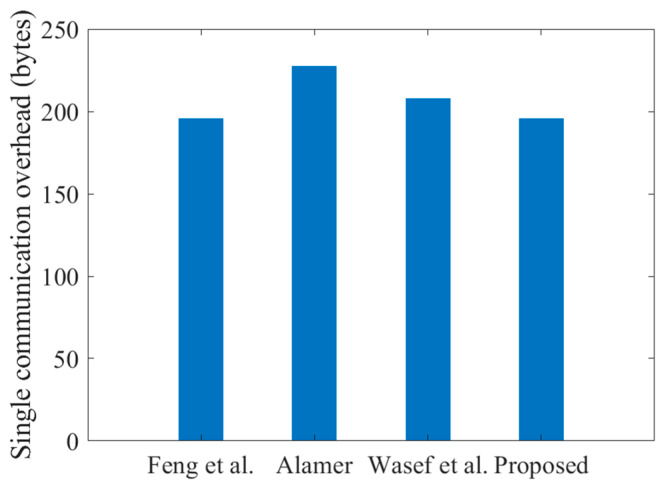
Communication overhead comparison in the unlinkable authentication scenario. Feng et al. [37], Alamer [24], Wasef et al. [12].

In the unlinkable authentication scenario, the UE needs to send an LEO signature of $3L_{G}+6L_{Z}$ = 144 bytes and plaintext of $2L_{ID}+L_{G}+L_{T}$ = 52 bytes. When the LEO completes authentication, 84 bytes of information will be returned. The signature information for the schemes of both Feng et al. [37] and Wasef et al. [12] was $3L_{G}+6L_{Z}$ = 144 bytes, while the schemes of Alamer [24] required $6L_{G}+6L_{Z}$ = 192 bytes. Since other SGS-based schemes have different architectures from the proposed scheme, we tried to unify their certification scenarios and situations for a better analysis. It was assumed that at least the ID and time stamp are required in these schemes. The trust value of the scheme of Feng et al. [37] was 4 bytes according to their article. A comparison of communication overhead from the UE to the access point is shown in Figure 10. As can be seen from the figure, the communication overhead of the proposed scheme was slightly smaller than the schemes of Alamer [24] and Wasef et al. [12], while it was equal to Feng et al. [37].

In batch authentication, the UE needs to send a $4L_{G}+2L_{ID}+L_{T}$ = 64 bytes message to the LEO, and the LEO returns a message of 84 bytes. A comparison was made with the communication overheads of the authentication protocol of Xue et al. [11] and Wasef et al. [12]. The results are shown in Table 5. According to the analysis results, the overall message length of the proposed scheme is shorter than that of the other related works.

Assuming that the plaintext length is 16n bytes (i.e., n is divisible by 16), then the ciphertext length is 16n bytes in AES encryption. If the plaintext length is 16n+m bytes and m<16, the ciphertext length is $16\left(n+1\right)$ bytes. In the roaming authentication phase, $K_{1}$ is key with a length $L_{r}$ = 16 bytes, and the size of $Ticket_{i}$ is $L_{ticket}=16*\left(2L_{ID}+L_{r}+L_{T}\right)/16$ = 64 bytes. Due to the use of symmetric encryption for key negotiation, we needed to calculate its communication cost separately. The size of $c_{1}$ sent by UE to LEO is $L_{c_{1}}=16\left(L_{r}+L_{ticket}\right)/16$ = 80 bytes, and the total message length is $L_{ID}+L_{G}+L_{c_{1}}+L_{H}+L_{T}$ = 152 bytes. The size of $c_{2}$ is $L_{c_{2}}=16*\left(L_{ID}+L_{r}\right)/16$ = 32 bytes, the message size sent by the LEO to the UE is $L_{ID}+L_{G}+L_{c_{2}}+L_{H}+L_{T}$ = 104 bytes. Despite the higher communication overheads of roaming authentication, it drastically reduces the computational latency, which is acceptable.

## 7. Conclusions

In this paper, we investigated on-demand access and roaming authentication protocols in a multi-operator heterogeneous scenario in a satellite–ground integrated network. We have proposed a scheme that includes anonymous unlinkable and batch authentication protocols, as well as fast roaming authentication. Specifically, users with higher privacy requirements are suitable for the unlinkable authentication scenario, where the scheme can provide anonymity and unlinkability; a large number of users with higher efficiency requirements are suitable for the batch authentication scenario, where the scheme provides traceable anonymity. In addition, for users who need to switch between multi-operator networks, this scheme provides a cross-domain fast roaming authentication solution. The proposed protocol delegates the authentication task to the satellite, which significantly reduces the transmission delay in the SGIN. We performed a formal analysis using ProVerif and an informal analysis to prove the security of the scheme. In addition, we evaluated the performance of the scheme and the results showed that our scheme is effective.

To enhance our research, we intend to study short group signature algorithms in more depth in our future work and propose more secure and more efficient protocols for unlinkability authentication scenarios based on zero-knowledge proofs. In addition, the application scenario of 6G will be more complex than 5G, and it is a challenge to cope with the fair billing of multiple operators and balance their interests. Especially, the introduction of eSIMs brings novel security risks and conflicts of interest. Thus, in the future, we will further consider the interests of multi-operator scenarios and try to find a suitable solution to authentication.

## Figures and Tables

**Table 1:** Summary of related works *.

Ref.	Performance Objective	Algorithm/Scheme	Scenario	Motivation
[2]	6G security architecture	-	6G	Research on 6G security requirements, analyzing 6G security architecture
[16]	access authentication	bilinear pairing	SIN	Research on group authentication using satellite broadcasting
[17]	unified authentication architecture	-	heterogeneous B5G	Research on a heterogeneous unified authentication architecture
[18]	access authentication	RLWE	post-quantum era	Research on access authentication against quantum cryptography
[19]	access authentication	bilinear pairing	SAGIN	Research on satellite multicast authentication protocols
[20]	access authentication	ECC	SIN	Research on secure access authentication protocols
[23]	group signature	SGS	-	Research on unlinkable group signatures
[12]	access authentication	SGS	-	Research on batch authentication scheme for SGS
[24]	access authentication	SGS	-	Research on signcryption scheme for SGS
[25]	batch authentication	ECC	VANET	Research on fast and anonymous batch and rebatch authentication
[26]	batch authentication	ECC	M2M	Research on lightweight group and rebatch authentication
[27]	access authentication	ECC	IoT	Research on authentication that quickly validates massive requests
[28]	access and handover authentication	secret sharing	multi-operator networks	Research on cross-domain handover authentication
[29]	access and roaming authentication	ECC and SBC	SGIN	Research on decentralized authentication and charging fairness
[30]	access and roaming authentication	SGS	SIN	Research on a user cross-domain roaming scheme
[31]	access and roaming authentication	ECC	SIN	Research on a secure roaming and key agreement scheme
[32]	access and handover authentication	CRT	HSR and SGIN	Research on efficient authentication and handover of terminals for railways

* SIN: space information network. B5G: beyond 5G. RLWE: ring learning with errors. SAGIN: space–air–ground integrated network. ECC: elliptic curve cryptography. SGS: short group signature. VANET: vehicular ad hoc network. M2M: machine to machine. IoT: Internet of things. SBC: smart billing contract. CRT: Chinese remainder theorem. HSR: high-speed rail. -: there is no relevant statement about this work.

**Table 2:** Comparison of operation time overheads.

Operation	Description	Time Overload (ms)
$T_{Pairing}$	A bilinear pairing $\hat{e}:G\times G\to G_{T}$	1.108615
$T_{Add_{1}}$	An addition operation a+b	0.000027
$T_{Add_{2}}$	An addition operation $g_{1}+g_{2}$	0.002787
$T_{Mul_{1}}$	A multiplication operation $ag_{1}$	0.882037
$T_{Mul_{2}}$	A multiplication operation $g_{T_{1}}g_{T_{2}}$	0.000949
$T_{Exp}$	An exponentiation operation $g_{T}^{a}$	0.148430
$T_{Hash_{1}}$	A hash function $\left\{0,1\right\}*\to Z_{q}^{*}$	0.000258
$T_{Hash_{2}}$	A hash function $\left\{0,1\right\}*\to G$	1.976653
$T_{Hash_{3}}$	A hash function $\left\{0,1\right\}*\times G\to Z_{q}^{*}$	0.030638

**Table 3:** Computational overloads in the unlinkable authentication scenario.

Scheme	Computational Overload (ms)	
	Signing	Verifying
Feng et al. [37]	$9T_{Mul_{1}}+5T_{Exp}=8.680483$	$13T_{Mul_{1}}+3T_{Pairing}+2T_{Exp}=15.089186$
Alamer [24]	$12T_{Mul_{1}}+2T_{Pairing}+2T_{Exp}+T_{Hash_{2}}=15.075187$	$8T_{Mul_{1}}+3T_{Pairing}+5T_{Exp}+T_{Hash_{2}}=13.100944$
Wasef et al. [12]	$11T_{Mul_{1}}+2T_{Pairing}+T_{Exp}=12.068067$	$10T_{Mul_{1}}+2T_{Pairing}+3T_{Exp}=11.48289$
Proposed	$9T_{Mul_{1}}+4T_{Exp}=8.532053$	$10T_{Mul_{1}}+2T_{Pairing}+3T_{Exp}=11.48289$

**Table 4:** Computational overload in the batch authentication scenario.

Scheme	Computational Overload (ms)	
	Signing	Verifying
Xue et al. [11]	$T_{Mul_{1}}+T_{Hash_{3}}=0.912675$	$\left(n+2\right)T_{Mul_{1}}+3nT_{Hash_{3}}$
Wasef et al. [12]	$11T_{Mul_{1}}+2T_{Pairing}+T_{Exp}=12.068067$	$\left(6n+7\right)T_{Mul_{1}}+2T_{Pairing}$
Proposed	$3T_{Mul_{1}}+T_{Exp}=2.794541$	$nT_{Exp}+T_{Pairing}$

**Table 5:** Communication overhead comparison in the batch authentication scenario.

Scheme	Message Length (Bytes)	
	UE to LEO	LEO to UE
Xue et al. [11]	$2L_{ID}+2L_{T}+2L_{G}+L_{Z}+2L_{ID}+L_{G}=144$	$L_{T}+2L_{ID}+3L_{G}+L_{Z}=100$
Wasef et al. [12]	$2L_{ID}+4L_{G}+6L_{Z}+L_{T}=166$	$2L_{ID}+L_{T}+L_{G}+2L_{Z}=84$
Proposed	$4L_{G}+2L_{ID}+L_{T}=64$	$2L_{ID}+L_{T}+L_{G}+2L_{Z}=84$

## Data Availability

Not applicable.
